# Effect of composite/amalgam thickness on fracture resistance of maxillary premolar teeth, restored with combined amalgam-composite restorations

**DOI:** 10.4317/jced.52726

**Published:** 2016-07-01

**Authors:** Maryam Firouzmandi, Maryam Doozandeh, Zahra Jowkar, Sanaz Abbasi

**Affiliations:** 1 DMD, MScD, Assistant Professor. Department of Operative Dentistry, School of Dentistry, Shiraz University of Medical Sciences, Shiraz, Iran; 2 Undergraduate Dental Student. Student Research Committee, School of Dentistry, Shiraz University of Medical Sciences, Shiraz, Iran

## Abstract

**Background:**

Combined amalgam-composite restorations have been used through many years to benefit from the advantages of both dental amalgam and composite resin. Two variations have been mentioned for this technique, this study investigated the fracture resistance of maxillary premolar teeth with extended mesio-occluso-distal (MOD) cavities, restored with the two variations of combined amalgam-composite restorations.

**Material and Methods:**

Sixty intact extracted premolar teeth were randomly divided into 6 groups (G1-G6) of 10 teeth. G1; consisted of intact teeth and G2; consisted of teeth with MOD preparations were assigned as the positive and negative control groups respectively. Other experimental groups after MOD preparations were as follows: G3, amalgam restoration; G4, composite restoration; G5 combined amalgam-composite restoration with amalgam placement only on 1mm of the gingival floor of the proximal boxes; G6, combined amalgam-composite restoration with amalgam placement to the height of contact area of the proximal surface of the tooth. Fracture strength of the specimens was measured and the data were analyzed using one-way analysis of variance (ANOVA). The level of significance was *P*<0.05. Fracture mode of the specimens was also recorded.

**Results:**

G1 had the highest value of fracture resistance (1736.90 N). G2 and G3 had the lowest fracture resistance (775.70 N and 874.70 N, respectively). The difference between G 4, 5 and 6 was not statistically significant. However, G4, G5 and G6 showed significantly higher resistance to fracture compared to G2 and G3. Fracture modes were favorable in all of the study groups except in G6.

**Conclusions:**

Fracture resistance of the premolars restored with the two variations of combined amalgam-composite restoration was similar to that achieved with composite restoration alone and more than that of amalgam restoration alone. It can be concluded that the thickness of amalgam in combined amalgam-composite restorations did not affect fracture resistance of the teeth.

** Key words:**Amalgam, composite, fracture resistance, restoration.

## Introduction

Numerous restorative options have been tried to restore extensively carious teeth to an optimum state of health, function, and aesthetics. However, the ideal method which requires minimum tooth removal during cavity preparation and simultaneously provides adequate strength and aesthetics is still a matter of debate. Among materials used to restore extensively carious teeth is dental amalgam, with proven clinically acceptable mechanical properties and serviceability in the oral environment through many decades (1); however, despite these benefits, it does not help strengthen the remaining tooth structure due to its high modulus of elasticity (2) and its inability to bond to the tooth structure (3,4). Moreover, to restore a tooth with amalgam, more extensive preparation of healthy structures is needed which can in turn make the reduced teeth more prone to fracture (5).

Another restorative material for these situations is composite resin which hopefully needs conservative cavity preparation that increases the fracture resistance of the teeth consequently (1). Moreover, these adhesive restorations can reinforce the remained tooth structure by better distributing the functional stresses across the bonding interface (6).

On the other side, polymerization shrinkage, difficulty of achieving tight inter proximal contacts in posterior teeth, wear at composite resin contact area and potential for microleakage especially when the gingival margin lies apical to the cementoenamel junction are among its drawbacks (7,8). Furthermore, gingival margin of proximal box of class II composite restoration is usually composed of aprismatic enamel or dentin. Bonding to both of these substrates is challenging due to altered etching pattern on aprismatic enamel (9), and the presence of tubular fluid and bond degrading matrix-metalloproteinase in dentin substrate (10,11). Moreover, bonding to deep gingival margins of Cl II cavities may be compromised because of inadequate moisture control. Accordingly, despite better initial composite resin marginal adaptation compared to amalgam, recurrent caries is more prevalent in composite restorations (9,12,13).

To overcome the drawbacks and to benefit from the advantages of both dental amalgam and composite resin, combined amalgam-composite restorations have been recommended by many researchers (1,8,14,15). Combined amalgam-composite restora-tions resulted in better proximal contacts and contours compared to composite restorations and better retention compared to amalgam restorations (1). Moreover a clinical study showed that combined technique performed excellently after 6.4 years (15). Less microleakage at the amalgam/cementum and amalgam/composite compared to composite resin/cementum interface was also reported (16). Combined amalgam- composite restorations compared to amalgam alone increased resistance to fracture in endodontically treated teeth (8).

Two variations have been mentioned for this technique. To the best knowledge of the authors no study compared the effect of the two combined amalgam-composite restoration variations on fracture resistance of teeth with extended mesio-occluso-distal (MOD) cavities. This study was designed to compare fracture resistance of maxillary premolar teeth with extended MOD cavities, restored with two variations of combined amalgam-composite restorations.

## Material and Methods

Following approval of the research protocol by the University Ethics Committee, sixty newly extracted sound maxillary premolars with similar crown sizes, free of caries, restoration and crack were selected and stored in 0.5% thymol solution at 4°C until use. A stereomicroscope (Carl Ziess, Oberkochen, Germany) at 20 X magnification was used to select teeth without any defect such as cracks. The selected teeth were mounted in cubic custom-made acrylic boxes (Acropars, Tehran, Iran) to the depth of 2mm below the CEJ (cemento-enamel junction) with the long axis of the tooth perpendicular to the base of the block and then randomly assigned to 6 experimental groups (G1-G6, n=10). All the procedures were done by the same operator. G1 (positive control) consisted of ten intact teeth. MOD cavities were prepared in the remaining five groups using No 008 diamond fissure burs (Dia. Tessin, Gordevio, Switzerland), at high speed, with water cooling with the occlusal depth of 2 mm, parallel facial and lingual walls, isthmus width of about one-half of the inter-cuspal distance, proximal box width of one-third of the total facio-lingual width of the tooth in height of contour area, and gingival floor of the proximal box extending 1 mm apical to CEJ. A digital caliper (Mitutoyo Digimatic, Mitutoyo, Japan) at 0.1-mm sensitivity was used to verify measurements for proper and accurate standardization of cavity dimensions (17). The bur was discarded after five preparations. G2 (negative control) consisted of teeth with MOD cavities without any restoration. In G3, after cavity preparation, the teeth were rinsed and gently dried. A matrix retainer system (Tofflemire, Miltex Inc, York, PA, USA) was fixed around the tooth. The first increment of a high copper admixed amalgam was placed in the depth of the proximal box and condensed by the most appropriate condenser. Further increments were condensed in a similar manner to fill the preparation. Then, the matrix band was removed. A moist cotton pellet was used to smooth the surface of the amalgam, which then was allowed set one hour at least. The teeth in G4 were restored with composite as follows. After cavity preparation, rinsing and drying, the tooth was etched with 37% phosphoric acid gel (Ivoclar, Vivadent, Schaan, Liechtenstein) for 30 seconds on enamel and 15 seconds on dentin, rinsed using an air-water spray for 20 seconds and gently air-dried. Two consecutive coats of Single Bond (3M ESPE, Dental products, USA) were homogenously applied over the surface and the solvent was evaporated with gentle air drying for 5 seconds and cured for 10 seconds according to manufacturer’s instructions with light curing unit (Diadent, Fast power program 800 mw/cm2, South Korea). Then a metal matrix band was placed around the tooth and resin composite (Z250, 3M ESPE Dental products, USA) was inserted in the preparation using the oblique incremental technique with the depth of no more than 1.5 mm for each increment. Each increment was cured for 20 seconds with the same light curing unit. A glycerin gel layer (Deox, Ultradent, South Jordan, UT, USA) was applied over the last increment of composite resin before curing to ensure complete polymerization in an anaerobic environment. After removing the matrix band, axial aspects of the restoration were cured again for an additional 60 seconds.

Kournetas *et al.* (14) and Geiger *et al.* (8) suggested two different variations of combined amalgam-composite restorations and the current study employed their methods in G5 and G6 respectively. In G5, after cavity preparation, rinsing, drying, and setting a matrix retainer system in place, 1mm of the gingival floor of the boxes was restored with admixed amalgam (GS-80, SDI Ltd, Melbourne, Australia). Surface mercury-rich amalgam layer was removed after condensation. After 5 minutes, the remaining cavity walls and amalgam surface were etched with 37% phosphoric acid gel (Ivoclar, Vivadent, Schaan, Liechtenstein) for 30 seconds on enamel and 15 seconds on dentin, and then rinsed and gently air-dried. The bonding agent (Single bond, 3M ESPE, Dental products, USA) was applied like the method described for G4. Then, the preparation was restored with composite resin (Z250, 3M ESPE, Dental products, USA) using oblique incremental technique as mentioned for G4. The procedures for the samples of G6 were similar to G5, except that the proximal boxes were restored with amalgam (GS-80, SDI Ltd, Melbourne, Australia), approximately at the level of the contact area of the tooth and the rest of the proximal boxes were restored with composite resin (Z250, 3M ESPE, Dental products, USA).

All samples were eventually subjected to continuous static compressive force (10 KN) using a universal testing machine (Zwick-Roell, Zwick, Ulm, Germany) at a vertical angle. The force was applied through a 5-mm-diameter round metal bar contacting only on the slopes of buccal and palatal cusps of each tooth and parallel to the long axes of the tooth. The cross head speed of the compressive force was 2 mm/min until fracture. Stress-strain curves and peak loads (in Newton) to fracture for each tooth were recorded. Statistical analysis of the data was performed by SPSS Version 17 software (IBM, Armonk, NY, USA) using one-way analysis of variance (ANOVA) and Tukey test. The level of significance was *P*<0.05.

After fracture of the specimens, failure modes were divided into two groups: favorable fractures in which the fractures stopped higher than 1 mm below the CEJ; unfavorable fractures in which the fractures stopped lower than 1 mm below the CEJ.

## Results

Fracture resistance in Newton (mean ± SD) for the six experimental groups is displayed in  Table 1. One-way analysis of variance (ANOVA) indicated that there were significant differences in fracture resistance among the experimental groups (*p*<0.05). The results of the Tukey test demonstrated that group 1 (intact teeth) had the highest value of mean resistance to fracture (1736.90 N) and group 2 (Cavity without restoration) and group 3 (Amalgam restoration) had the lowest fracture resistance (775.70 N and 874.70 N, respectively). The difference between groups 4, 5 and 6 was not statistically significant. However, groups 4, 5 and 6 showed significant higher resistance to fracture compared to groups 2 and 3.

**Table 1:**
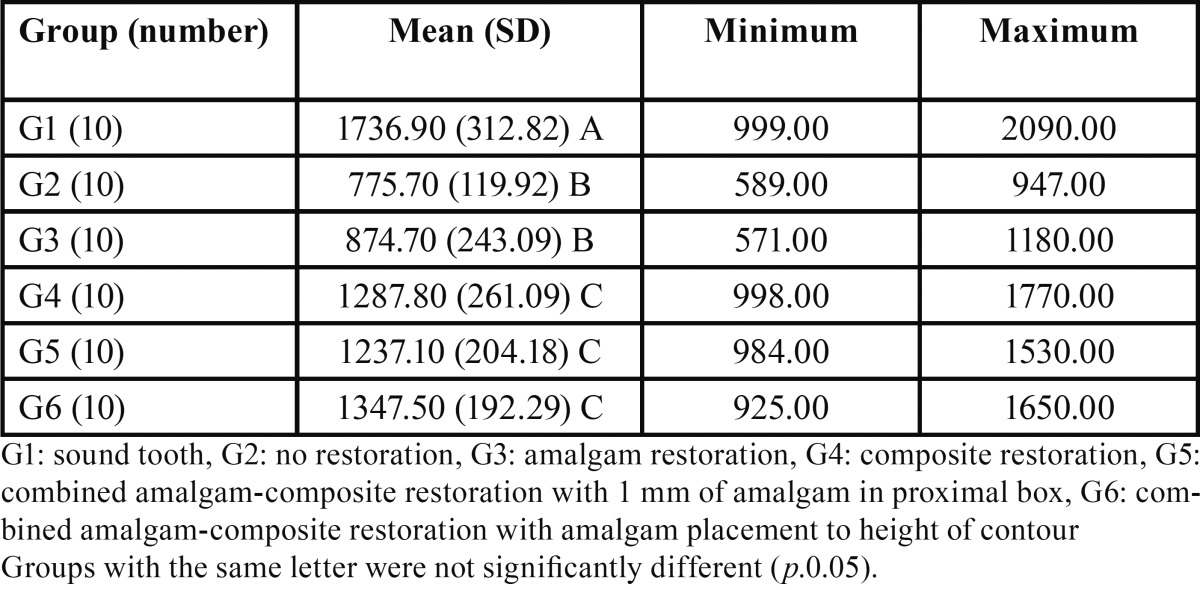
Fracture resistance in Newton for the study groups.

Failure modes are presented in  Table 2. Most of the fractures in G1, 2, 3, 4 and 5 were favorable. In G6 equal numbers of favorable and unfavorable fracture modes were recorded.

**Table 2:**
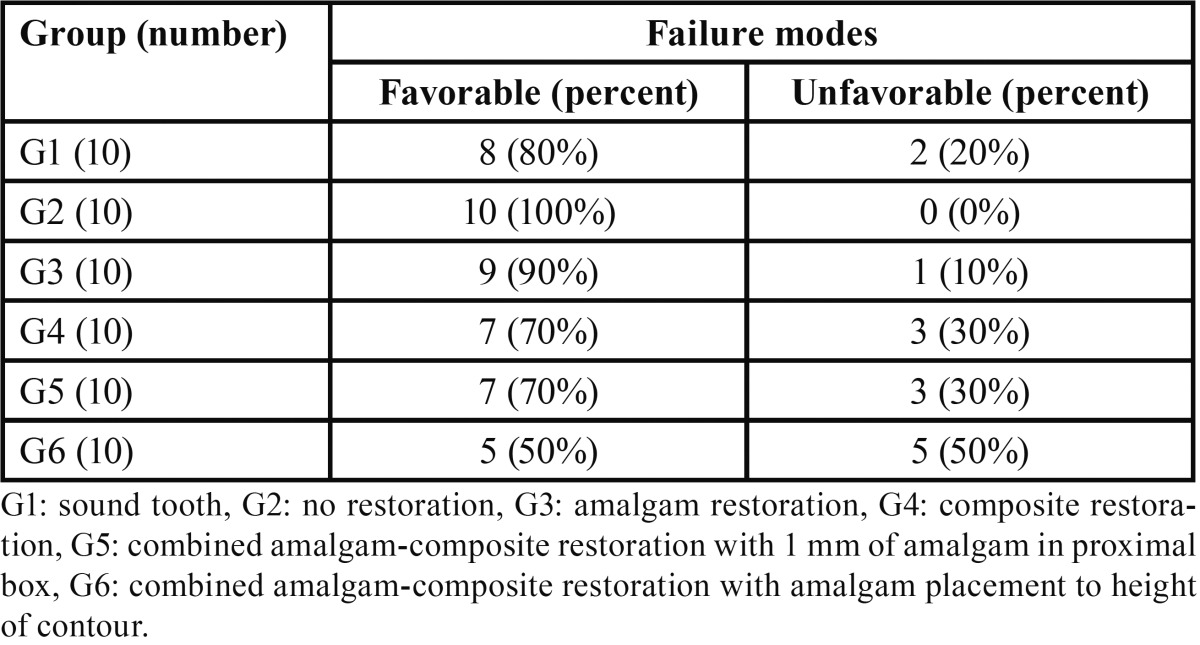
Failure modes of the study groups.

## Discussion

The results of the present study showed that fracture resistance of the premolars restored with the two variations of combined amalgam-composite technique was not significantly different, and both of them reinforced the remained tooth structure as with composite restoration. The combined amalgam-composite restorations are propounded as an alternative for amalgam repair with a more conservative method or as a means to provide more resistance to cusp fracture of the teeth with extended Cl II cavities (18). It is proposed that adhesive restorative materials reinforce the remaining tooth structure by enhancing structural continuity between buccal and lingual cusps (1,4). It seems that composite placed in occlusal portion, above the contact area in G6 was sufficient to exert splinting action. So, this version of the combined technique can be suggested in challenging clinical situations such as in cases of extensive proximal boxes with cervical margins located near or beneath the cementoenamel junction. This would provide aesthetic appearance of composite resin together with tight proximal contact and good marginal seal achieved by amalgam without compromising fracture resistance of the restored tooth.

On the other hand, increased bulk of composite in G4 and G5 could increase polymerization shrinkage stresses which might predispose the tooth to crack propagation and fracture. These stresses might be relieved through incremental placement of the composite in the current study.

Placement of a layer of amalgam under the composite in proximal box of class II composite restorations may reduce the amount of polymerization shrinkage stress through the resultant decreased bulk of the composite resin. Because of the difficulty in providing adequate isolation and light intensity in deep proximal boxes, marginal sealing of the bonded composite restorations might be compromised in these regions (19). Some improvements in dentinal marginal sealing of class II composite restorations with amalgam placement in the gingival third of the proximal box have been reported (14). Despite the absence of any chemical interaction between the two materials, an excellent marginal seal and better performance of the amalgam/composite resin interface compared to composite–tooth or amalgam-tooth interface were reported in the study by Tolidis *et al.* (16). They considered combined amalgam-composite restoration as an aesthetic and biologic alternative to conventional composite or amalgam restorations (16). Furthermore a recent *in vivo* study demonstrated better retention, contour and contact for combined restorations compared to conventional composite resin or amalgam restorations (1). According to the results of the current study, any type of the assessed combined amalgam-composite restoration can partly compensate for the reduced fracture resistance of the prepared tooth the same as the bonded composite restoration alone and it was more than the amalgam restoration alone. These findings are in line with some previous studies which showed the effect of bonded composite resin restorations in partial recovery of the fracture resistance of the prepared teeth (12,20). Failure mode was predominantly favorable in study groups except in G6. The specimens in G4, G5 and G6 resisted higher load until fracture compared to G2 and G3. The high fracture load together with the absence of reinforcing effect of composite resin in cervical region in G6 might cause fractures extending below the CEJ.

In the present study, bonding procedures for composite insertion were done only 5 minutes after amalgam condensation ignoring the negative effect of high surface tension of fresh amalgam on surface wettability. The aim of this approach was to simulate clinical condition, save time, and eliminate the need for temporary restoration placement. However, evaluation of the effect of delay between amalgam insertion and composite placement in combined technique seems to be necessary in future studies. Maximum generated biting force in the premolar region by a patient with no parafunctional habits usually does not exceed 300-400 N (21). All of the restorative techniques in this study resulted in fracture resistance values more than the aforementioned normal biting force in premolar region. In the present study, loading direction parallel to the longitudinal axis of the teeth might result in a more uniform stress distribution, which is different from dynamic fatigue loading typical of mastication, with a mixture of shear and compressive forces.

The effects of amalgam surface irregularities and entrapment of air between amalgam and composite on stress distribution, composite-amalgam bonding, and fracture resistance of combined restorations are not fully understood and need to be more investigated. Moreover, more information may be provided by studies including artificial aging, such as thermocycling or chewing stimulator.

To imply the results of this study to clinical situations and predict the longevity of the combined amalgam-composite restorations, *in vitro* studies with accurate simulation of the clinical conditions and long-term clinical trials are suggested.

## Conclusions

1) Fracture resistance of maxillary premolars with extended MOD preparations restored with two variations of combined amalgam-composite restoration with amalgam to the level of proximal height of contour or only at 1 mm of the gingival floor of the proximal box was similar to that achieved with composite restoration alone and more than that of amalgam restoration alone. So, the resultant reduced composite thickness in combined amalgam-composite restorations did not affect fracture resistance of the teeth.

2) The resistance of maxillary premolars to fracture was reduced significantly after MOD preparation.

3) None of the restorative techniques used in this study including amalgam restoration alone, composite restoration alone, and combined amalgam-composite restorations could completely restore the fracture resistance of the maxillary premolars with extended MOD preparations.
